# Supplementary material for: Venomix: a simple bioinformatic pipeline for identifying and characterizing toxin gene candidates from transcriptomic data
Source: PeerJ. 2018 Jul 31;6:e5361. doi: 10.7717/peerj.5361 (PMC6074769; doi:10.7717/peerj.5361)
Supplement: Supplemental Information 3 [file peerj-06-5361-s003.gz › FinalOutput_E-6/Neprilysin-1_1/finaltree.pdf]

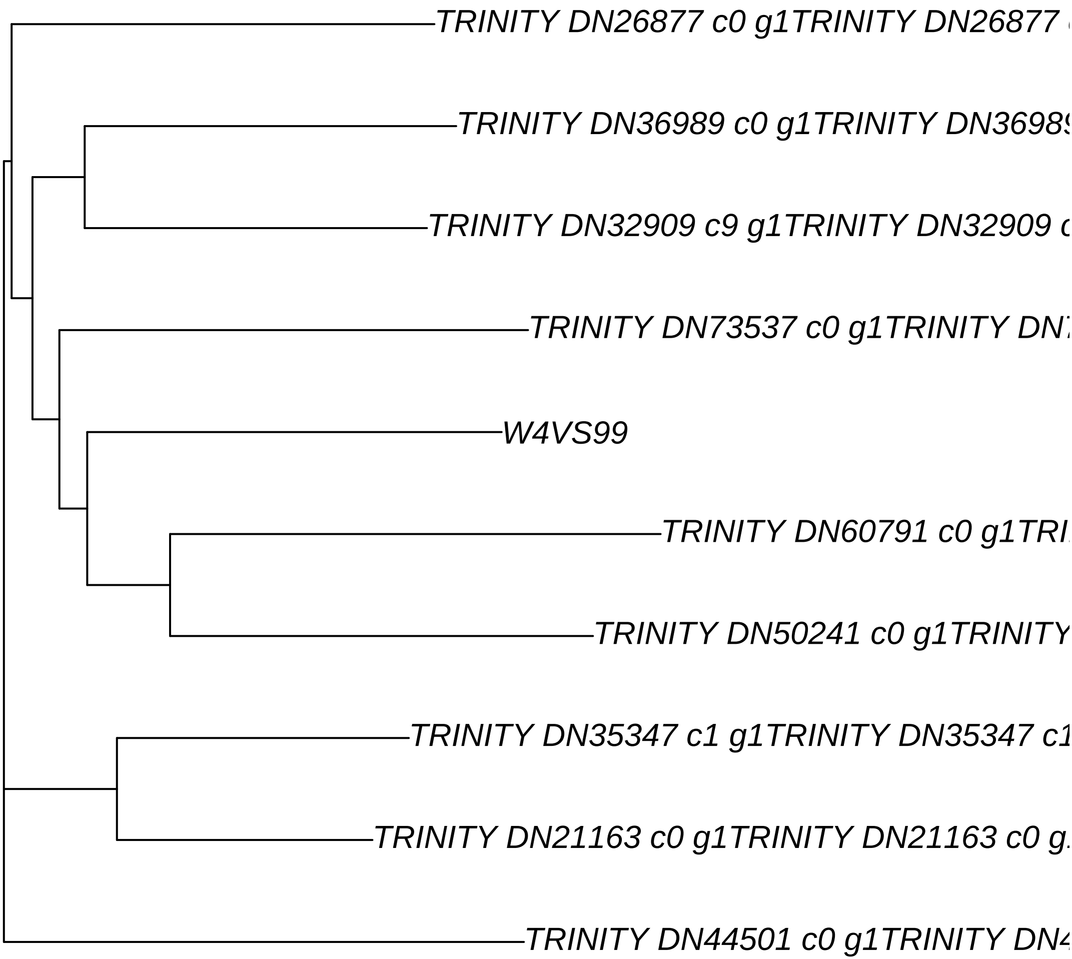
TRINITY DN26877 c0 g1TRINITY DN26877
TRINITY DN36989 c0 g1TRINITY DN3698
TRINITY DN32909 c9 g1TRINITY DN32909
TRINITY DN73537 c0 g1TRINITY DN
W4VS99
TRINITY DN60791 c0 g1TRI
TRINITY DN50241 c0 g1TRINITY
TRINITY DN35347 c1 g1TRINITY DN35347 c1
TRINITY DN21163 c0 g1TRINITY DN21163 c0 g
TRINITY DN44501 c0 g1TRINITY DN4
